# Might patients with cerebellar ataxia benefit from the Computer Assisted Rehabilitation ENvironment (CAREN)? A pilot study focusing on gait and balance

**DOI:** 10.3389/fbioe.2024.1385280

**Published:** 2024-06-24

**Authors:** Mirjam Bonanno, Paolo De Pasquale, Cristiano De Marchis, Antonino Lombardo Facciale, Giuseppe Paladina, Bartolo Fonti, Angelo Quartarone, Rocco Salvatore Calabrò

**Affiliations:** ^1^ IRCCS Centro Neurolesi Bonino-Pulejo, Messina, Italy; ^2^ Department of Engineering, University of Messina, Messina, Italy

**Keywords:** CAREN, cerebellar ataxia, neurorehabilitation, virtual reality, gait analysis

## Abstract

**Introduction:** Ataxia is a neurological symptom that causes decreased balance, loss of coordination, and gait alterations. Innovative rehabilitation devices like virtual reality (VR) systems can provide task-oriented, repetitive and intensive training with multisensorial feedback, thus promoting neuroplastic processes. Among these VR technologies, the Computer Assisted Rehabilitation ENvironment (CAREN) associates a split belt treadmill on a 6-degrees of freedom platform with a 180° VR screen and a Vicon motion capture system to monitor patients’ movements during training sessions.

**Methods:** Eight patients affected by cerebellar ataxia were enrolled and received 20 sessions of CAREN training in addition to standard rehabilitation treatment. Each patient was evaluated at the beginning and at the end of the study with 3D gait analysis and clinical scales to assess balance, gait function and risk of falls.

**Results:** We found improvements in kinematic, kinetic, and electromyographic parameters (as per pre-post- CAREN training), as well as in clinical outcomes, such as balance and risk of falls in ataxic patients. In addition, we found that trunk rotation improved, after CAREN intervention, approximating to the normative values.

**Discussion:** Our results suggested that CAREN might be useful to improve specific biomechanical parameters of gait in ataxic patients.

## 1 Introduction

Ataxia is a neurological symptom that causes alteration in locomotion, decreased balance, loss of coordination, dysmetria, action tremors and hypotonia. It often results from cerebellum damage for either genetic or acquired brain injury due to vascular and/or hemorrhagic lesions, and/or brain tumours, often located in the posterior cranial fossa (K and Kishore, 2020). The worldwide prevalence of cerebellar ataxia (CA) ranges from 2 to 43 cases per 100.000 population (Zotin, 2022). Physiologically, the cerebellar cortex has a pivotal role in initiating purposeful movements. During such movements, proprioceptors continually inform the cerebellum about the changing positions of muscles and joints. Given that, the cerebellar cortex compares intended movements with the current action and sends feedback signals to the motor cortex to adjust the activity of skeletal muscles. It smooths and coordinates complex sequences of skilled movements and regulates posture and balance. For instance, lesions involving midline cerebellar structures may cause gait and trunk impairments, whereas hemispheric lesions lead to homolateral limb ataxic symptoms (Cabaraux et al., 2023). In particular, gait pattern in CA is characterized by reduced gait speed and cadence, reduced step length, stride length and swing phase, increased base width, stride time, stance phase and double limb support phase (Serrao and Conte, 2018; Bonanno et al., 2023). These gait alterations are considered compensatory movements due to trunk instability, whereas the uncoordinated muscle activation is related to the actual cerebellar damage (Buckley et al., 2018).

Physiotherapy is the main treatment for gait and balance alterations for CA patients, although few authors have reported its effectiveness (Chien et al., 2022). Promising conventional rehabilitation approaches include dynamic balance training, customized interventions targeting balance and independence in activities of daily living, the Bobath approach, and personalised gait training (Kelly and Shanley, 2016; Yap et al., 2022). To date, new technologies are becoming popular in the neurorehabilitation setting. In particular, an emerging field of research is the use of virtual reality (VR) and augmented reality as therapeutic and rehabilitation approaches (Bogaert et al., 2023). VR technologies can provide task-oriented, repetitive and intensive training with multisensorial feedback, thus promoting neuroplastic processes (Bonanno et al., 2022). Indeed, the use of VR during rehabilitation sessions increases patients’ motivation and attention and allows the analysis of movements during training sessions (Cano Porras et al., 2019). Three different types of VR systems, i.e., non-immersive, semi-immersive and immersive are recognized in the clinical setting. The non-immersive and semi-immersive VR systems use a screen to display the environment with reduced level of immersion and presence. On the other hand, immersive VR systems consist of full integration of the user into the virtual environment, which provides sensory inputs to the patient. Among the immersive VR devices, the Computer Assisted Rehabilitation ENvironment (CAREN) (Motekforce Link, Amsterdam, Netherlands) combines a split belt treadmill on a 6-degrees of freedom platform with a 180° VR screen and a Vicon motion capture system to monitor patients’ movements during training sessions (Maggio et al., 2023). This innovative system has been already used to train other neurological conditions like multiple sclerosis (Kalron et al., 2016), Parkinson’s disease (Calabrò et al., 2020; Formica et al., 2023) and Huntington’s disease (Cellini et al., 2022). Nonetheless, the available literature about the use of VR in patients with CA is still limited to a few studies (Peri et al., 2019; Takimoto et al., 2021). Unlike conventional rehabilitation methods, VR has the main advantage of providing multisensorial visual and auditory stimuli, in a controlled and safe environment. Additionally, VR promotes motivation and engagement during rehabilitation, providing intensive, repetitive and task-oriented training. Therefore, patients can perform ADL (cooking, driving, etc.) without any risk, in simulated scenarios, through different and controlled levels of difficulty for each task. These aspects could have a role in promoting functional and motor recovery in CA patients as well as per other neurological disorders. In fact, unlike other neurological disorders in which the effectiveness of VR has already been demonstrated (Lacorte et al., 2021), the use of VR in CA patients has been less investigated as well as its role in inducing gait biomechanics changes.

In this pilot study, we aimed at evaluating the feasibility of VR training in a CAREN system to improve gait function for patients affected by CA.

## 2 Materials and methods

### 2.1 Study design and population

Eight ataxic patients (two males and three females) with the mean age of 61.3 and the standard deviation (SD) of 9.15 (see Table 1 for more details) participating to rehabilitation programs at the IRCCS Centro Neurolesi “Bonino-Pulejo”, in Messina (Italy), between June 2022 and May 2023, were enrolled in this pilot study.

**TABLE 1:** Socio-demographic and clinical data of the CA subjects included in the study.

	Age	Gender (females, males)	Education (years)	Time since injury (years)	Clinical characteristics	Aetiology
All patients	56.8 ± 10.6	5M; 3F	13 ± 4.3	9.6 ± 6.4		
Patient 1	48	M	13	9	Lower limb ataxia with mild right homolateral paresis and hypoesthesia	Posterior fossa astrocytoma
Patient 2	63	M	13	6	Trunk ataxia with left paresis	Ischemic stroke
Patient 3	56	M	18	5	Trunk ataxia with right paresis	Ischemic stroke
Patient 4	75	F	18	6	Lower limb ataxia with mild left homolateral paresis	Ischemic stroke
Patient 5	45	M	8	11	Lower limb ataxia with mild homolateral left paresis	Arteriovenous malformation
Patient 6	66	F	8	8	Lower limb ataxia with left paresis	Haemorrhagic stroke
Patient 7	60	M	8	7	Trunk ataxia with left paresis	Ischemic stroke
Patient 8	42	F	18	25	Trunk ataxia with left paresis and hypoesthesia	Posterior fossa ependymoma

All patients were in the subacute/chronic phase of the disease (from 2 to 6 months after the event) and all of them were referred to our specialized institute by other centers, where they were submitted to conventional physiotherapy alone without important improvement on ataxic gait.

Inclusion criteria were: 1) clinical diagnosis of CA on neurological and radiological examination, due to trauma, brain tumour, stroke or arteriovenous malformation; 2) age 18–75 years; 3) patients able to walk without assistance (score at Functional Ambulation Classification [FAC] ≥ 2). Patients were excluded if they had 1) cognitive, visual or auditory deficits that impair the comprehension and/or the execution of the listed exercises; 2) presence of unstable medical conditions (e.g., hypertension or hypotension, cardio-respiratory failure, etc.); 3) the absence of informed consent. Furthermore, exclusion criteria related to CAREN technology, including: 1) weight ≤20 kg (44 lb) or ≥135 kg (300lb), 2) FAC score of one or lower (need for continuous supervision by a person to help them maintain balance and move); 3) patients who are unable to adjust the safety sling correctly because of body shape, such as in pregnancy, colostomy bags, skin lesions that cannot be adequately protected, or for any other reason that prevents the sling from being adjusted correctly and painlessly, were also considered.

All experiments were conducted according to the ethical policies and procedures approved by the local ethics committee (IRCCS-ME-23/2022). All participants gave their written informed consent.

### 2.2 Procedures

In this pilot study, we enrolled eight patients affected by acquired CA who were able to walk independently. All patients received experimental rehabilitation treatment using the innovative CAREN system, in addition to conventional physiotherapy. Patients were trained for three-four times a week for 20 sessions, each session lasting about 45 min. The therapeutic protocol was tailored on the patients’ need, personalizing the setting and the difficulty of proposed exergames on patients’ motor resources, and it was oriented to the recovery of balance ability and of a correct locomotion pattern. Each session included a random sequence of the 5 VR scenarios, i.e., boat, microbes, active balance, traffic jam, step on it (for details see Table 2), with a 1–2-min break between the exercises. During all training sessions, patients wore a safety harness while a physical therapist stood beside and/or behind him/her to protect from falling. Notably, the rehabilitation treatment was performed in a dedicated space, next to the main clinical Centre, called “Innovation Neuro-Motion Lab” (where the CAREN is also located) in order to standardise both evaluation and training sessions. Conventional training sessions were instead performed in the traditional rehabilitation gym of the same building and included stretching and exercises to improve gait functions through weight shifting between lower limbs, stepping training over levels, heel strike/limb-loading acceptance, and push-off/initial swing of the moving limb.

**TABLE 2:** Description of CAREN scenarios and exercises used to train CA patients.

VR scenario	Description	Exercise goal
Boat	The setting was a marine environment with buoys to avoid; the patient guided the boat with his body until the final goal. The user’s task is to steer the boat by tilting their trunk laterally or using the center of pressure (CoP)	The goal is to maintain balance while the platform undergoes oscillations related to the boat’s movement and wave intensity
Microbes	The scenario is represented by the appearance of red-coloured viruses and green-coloured targets, respectively to be avoided and caught by means of a circular-shaped figure that follows the movement of the subject’s body. The subject is free to move over the entire area of the moving platform at a speed set by the physiotherapist	This application provides multiple challenges with different motor tasks, like moving within the area of the platform to avoid red-coloured viruses and catch green-coloured targets or moving backwards and forward around a green band in which the patient have to protect himself from an invasion by red-coloured viruses in the rest of the scenario. The physiotherapist can change the speed of the platform and the level of difficulty of the various tasks required
Active Balance	A maze in which the patient drove a red ball, moving the load up to the finish line. The scenario is characterized by a sphere representing the subject’s center of pressure (CoP). At the beginning of the training, the sphere is positioned in front of the entrance of a maze. To move the sphere along the maze, the subject leans forward and backward, left and right without moving their feet from their initial position	This exercise is designed for antero-posterior (AP) and medio-lateral (ML) balance training and load force modulation and control. The aim of the application is to finish the maze as quickly as possible without hitting the walls. The physiotherapist has the option of deciding the sensitivity of the footplate’s response to the loading force by the patient
Traffic jam	The setting is a crossroads with the patient driving a car	The subject has the objective of passing cars coming from the left by raising his left leg and cars coming from the right by raising his right leg. The physiotherapist can define the difficulty of the training by modifying different parameters
Step on it	In this exercise scenario, the subject walks on an infinitely long road seen from the top. During the walk, step length and width are measured, and the subject’s footprints appear on the screen to provide real-time feedback to the subject	The goal of this exercise is to encourage the subject to adjust their step length and width (step frequency) according to parameters set by the therapist

Gait analysis and motor clinical assessment were administered by a skilled physiotherapist (G.P.) at the beginning (pre) and at the end (post) of training sessions, through specific motor outcomes and instrumental gait analysis by using BTS Gaitlab. The assessor was different from the physiotherapist (A.L.F.) who provided the CAREN training sessions, in order to reduce biases.

**FIGURE 1:**
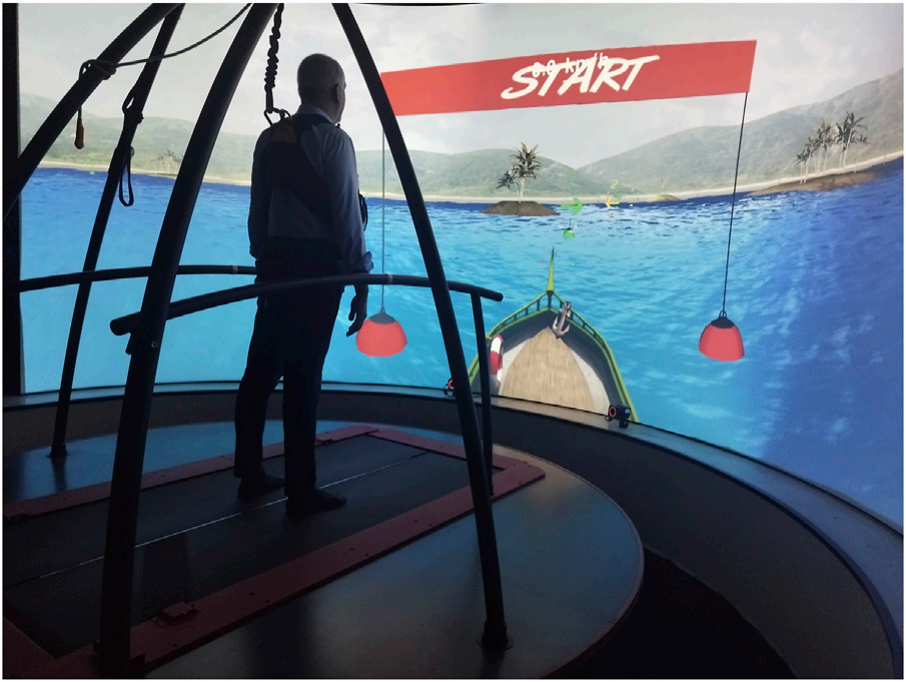
Shows a CA patient during a training session on the CAREN system performing “Boat” exercise.

The VR system used by the CA patients was the CAREN (Motekforce Link, Amsterdam, Netherlands). This system consists of an electro-hydraulic 3 m diameter motion platform (Rexroth Hydraudyne, MOTEK, Micro motion) that can be manipulated by 6 degrees of freedom (x-y-z translation and pitch-roll-yaw rotation). The platform is equipped with force plates under a double-banded treadmill that can reach a speed of 5 m/s. The platform movement is either driven by the patient’s movement or preprogrammed in synchrony with function curves that define a specific pathway in the virtual environment. In addition, the CAREN is equipped with a 180° screen that provides different degrees of VR immersion. Indeed, the system offers different combinations of sensory feedback, including visual, auditory, vestibular and tactile stimuli. During the rehabilitation sessions with the CAREN, the CA patients were harnessed with a body safety vest attached to an overhead truss, allowing patients to move freely on the treadmill. They were also asked to remove their shoes before stepping onto the platform to enhance proprioceptive feedback (Figure 1).

The rehabilitation program consisted of different virtual exercises in immersive scenarios, which are reported on Table 2.

Although the CAREN system is also an advanced tool for performing accurate motion analysis (using infrared cameras that detect markers placed on different parts of the subject’s body), the gait analysis of this study was carried out in a specific instrumental gait analysis system, using the BTS Gaitlab (see the description at 2.4).

### 2.3 Clinical outcomes

A skilled physiotherapist (G. P.) assessed the patients at pre- and at post-treatment using the following clinical scales/tests: 1) Berg Balance Scale (BBS) (Miranda-Cantellops and Tiu, 2024) which consists of 14 items, scored from 0 to 4, evaluating static and dynamic balance tasks; 2) 6-Minutes walking test (6MWT) in which the patient is asked to walk as fast as possible in 6 minutes, slowing down his or her gait or stopping whenever he or she feels necessary if fatigued (Agarwala and Salzman, 2020); 3) Timed up and go (TUG) which consists of timing how long it takes a person to get up from a chair, walk 3 m, turn around and then sit down again, and it gives an estimation of risk of falls (Browne and Nair, 2019); 4) 10-m walk test (10MWT) is used to assess walk speed in m/s over a short distance and the final score is given from the average of three trials (Peters et al., 2013); 5) Falls efficacy scale-International (FES-I) (Caronni et al., 2022) is a 16-items measure of fear of falling, it ranges from a minimum of 16 (no fear/concerns of falling) to a maximum of 64 (strong concern about falling); 6) Tinetti scale (TS) is a 16 items (7 for gait and nine for balance) measure in which a total score ≥19 is indicative of high risk of falls while a total score between 19 and 24 indicates a moderate risk of falls (Scura and Munakomi, 2024).

### 2.4 Instrumental gait analysis

**FIGURE 2:**
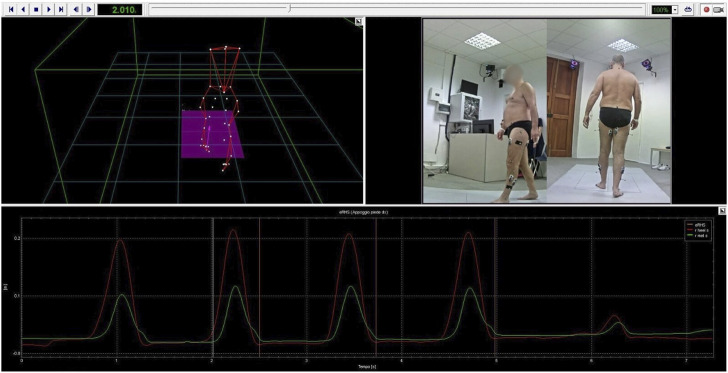
BTS SMART-Clinic software. Figure shows a data processing procedure for one of the participants. The 3D human body model reconstructed from the optoelectronic system as well as the events detected from the force platform and the video recorded during the gait trial are shown.

A skilled physiotherapist (G.P.) together with a biomedical engineer (P.D.P.) evaluated the gait cycle of the patients at pre- and at post-treatment by using the BTS Gaitlab (BTS Bioengineering, Milan, Italy). This system is a comprehensive gait analysis system comprising fully integrated instrumentation for objective and quantitative clinical assessment (see Figure 2). This instrumental evaluation allows both clinicians and physiotherapists to have an objective idea on posture and gait alterations, load anomalies and muscle deficits, which cannot be pointed out by the traditional clinical scales/tests.

To accomplish this purpose, it uses.

• 8 infrared cameras (BTS SMART-DX)
• 4 sensor platforms (BTS P-6000)
• 8 wireless electromyography probes (BTS FREEEMG 1000)

The BTS SMART-Clinic software included in the BTS GAITLAB system, provides libraries containing the main scientifically validated analysis protocols (Kadaba et al., 1989; Davis et al., 1991). In particular, the protocol used in our study for the gait analysis was the “DAVIS Heel: multifactorial gait analysis”. This was developed to provide quantitative and objective data needed to study human locomotion kinematics, kinetics and associated muscles electrical activity to evaluate gait functionality. The “Newington marker set” used in Davis protocol (Davis et al., 1991), which introduced a particular data collection technique for gait analysis, inspired the implementation of the protocol. This protocol requires the measurement of the subject’s anthropometric parameters, such as: weight, height, tibia length, distance between the femoral condyles, knee diameter, ankle diameter, distances between the anterior iliac crests and pelvis thickness. Then, the optoelectronic system allows to perform kinematic analysis detecting the exact position of the markers placed on the patient’s body and through an appropriate software calculates the flexion-extension, abdo-adduction, extra-intra-rotation angles of the hip, knee, pelvis, trunk and ankle joints from these points. Gait cycle events, such as foot initial ground contact and lifted off the ground events, can be automatically identified either from markers data or from force platforms data. Therapists verify the automatic software events identification.

The first detection (standing phase) is obtained from asking the patient to maintain an indifferent standing position for 5 s. After that, the patient is asked to walk at a speed he/she considers normal, starting from a point which is set when his/her feet are on each force platform of the Gaitlab. After some acquisitions, usually 6 (number of strides for each acquisition, mean value ±SD = 2.5 ± 0.6), that are considered sufficient in terms of measurement repeatability the baseline evaluation is concluded. During the gait analysis, it is also possible to detect electromyography (EMG) activity through the 8 wireless electromyography probes (4 for each limb), connected to the Smart Analyzer system (Version 1.10.469.0; BTS, Milan, Italy). In our study, we evaluated EMG (FREEEMG 1000 system; BTS Bioengineering, Milan, Italy) signals from the following muscles: gastrocnemius lateralis, tibialis anterior, rectus femoris, and semitendinosus (Stegeman and Hermens, 2007). Skin was carefully prepared (i.e., cleaning, and drying) for the positioning of bipolar adhesive surface electrodes. After this procedure, the electrodes were positioned over the muscle belly, aligning with the direction of muscle fibers as per the European guidelines for surface electromyography (SENIAM) (Stegeman and Hermens, 2007).

The healthy adults’ normative bands (including 40 normal healthy subjects, 28 males and 12 females, with age range of 18–40 years) (Kadaba et al., 1990; Materia, 2023) relative to the kinematics, kinetics and EMG activity are provided to evaluate eventually gait dysfunctionality.

Lastly, the following information obtained from BTS Gaitlab were processed during the offline analysis.

• *Kinematics:* spatio-temporal parameters, pelvic obliquity, and trunk rotation angles are measured during standing acquisition and kinematic analysis evaluated from the walking trials recordings.

Spatio-temporal parameters and normative data were provided by BTS Gaitlab for each patient and for each evaluation session. Temporal parameters as gait stance and swing time expressed in seconds (s) and gait cycle single support and swing normalized on gait stride (%), together with the spatial parameters as step width (m) and the gait velocity (m/s) were averaged across all cycles for each subject for each evaluating session (pre and post). Pre-treatment data were compared to the normative values, to underline the gait deficit. Right and left limbs data were compared separately at pre- and post-intervention. In addition, the shape symmetry index R_CIRC_ (Ambrosini et al., 2016) was computed to compare the ataxic and healthy groups in terms of joints trunk rotation angles, and pelvic obliquity to quantify the dysfunctionality of gait, providing an estimation of the difference between the pathologic gait and the physiological one.

$$R_{CIRC}=\frac{C_{xy}}{\sqrt{\sum_{n=1}^{101}{x\left(n\right)}^{2}\sum_{n=1}^{101}{y\left(n\right)}^{2}}}$$ (1)

In which x is the waveform related to the ataxic subjects, while y corresponds to the average waveform of the healthy population. C_xy_ is the circular cross-correlation function at lag 0. R_CIRC_ ranges from −1 to 1 (i.e., identical amplitude profiles shape).

• *Kinetics:* moments and powers at the hip, knee and ankle joints.

The shape symmetry index R_CIRC_ (Eq. 1) of the dynamic results concerning the joint moments, powers, and ground reaction forces was computed to compare the pathological and healthy groups in terms of joint moments, powers, and forces. Joint moments and powers, normalized by the subject’s weight (N*m/kg and W/kg), as well as ground reaction forces evaluated as percentage of body weight, were averaged across all cycles for each subject at each evaluating session (pre and post). Right and left limbs were reported separately.

• *Electromyography:* muscle activation and deactivation.

The EMG signals associated with muscle contraction were recorded with surface electrodes. The raw signals (millivolts) were filtered with a band pass filter (20–450 Hz) and time-normalized for the duration of the gait cycle (% gait cycle). Signal amplitude, which is proportional to the force expressed by the muscle, was analyzed through the calculation of the square root of average power of the signal (Root mean square–RMS) in moving windows selected as 10% of the gait cycle (Reaz et al., 2006).

The simultaneous contraction of agonist and antagonist muscles during gait provides support, balance, propulsion and improves movement efficiency. Muscle co-contraction estimation provides a useful tool to understand the effect of a disorder on muscle strategy control. To this end, to estimate the co-contraction or co-activation index we used the method in (Eq. 2) (Souissi et al., 2017) in which the norm EMG antagonist (t) was the lower normalized EMG and norm EMG agonist (t) was the higher value.

$$CoAct\left(t\right)=\frac{2\times norm\;EMG\left(t\right)antago}{\left(norm\;EMG\left(t\right)antago+norm\;EMG\left(t\right)ago\right)}\times 100$$ (2)

In which the antagonist and/or agonist were the Tibialis Anterior-Gastrocnemius lateralis and Rectus femoris-Semitendinosus muscle pairs.

Furthermore, we conducted the same analyses based on patients’ clinical characteristics, comparing the affected and unaffected sides for each evaluated parameter.

### 2.5 Statistical analysis

The dependence of overall kinematic, dynamic and EMG instrumental outcomes on experimental factors was analysed with a linear mixed model (LMM) that accounts for interindividual variability by including the participant as a random effect. The session (S, pre and post), the laterality (L, right and left side) and impairment (I, affected and unaffected side) were treated as fixed effect factors. The experimental factors (E) were treated as a fixed effect with categorical (dummy) variables. Data were fitted independently for each experimental factor with the model described in Eq. 3.

$$Y=u_{0}+\alpha_{0}E+\epsilon$$ (3)

In Eq. 3, u_0_ represents the individual intercept and accounts for inter-individual differences. The coefficient α_0_ represents fixed-effects; thus, the modulation of the response variable by the main factors respectively S, L and I. The estimation of model parameters was based on the maximum likelihood approximation. To test the significance of each fixed effect term in the selected model, a hypothesis test on the fixed effect terms applying analysis of variance (ANOVA) on the fitted LMM was performed.

Continuous variables were expressed as mean and SD values, whereas categorical variables (i.e., education) were expressed as frequencies and percentages. The normal distribution of the sample was investigated through Lilliefors test. According to the normality of all variables, we chose a parametric analysis. The Student’s t-test for paired samples was used to compare overall pathological subjects’ parameters with healthy normative values. All the analyses were implemented in Matlab (MATLAB (R2022a), Natick, Massachusetts: The MathWorks Inc.; 2022).

## 3 Results

All participants completed the rehabilitation program without reporting side-effects related to VR, including cybersickness (i.e., headache, dizziness, and nausea), maybe thanks to the synchronization between visual feedback and platform’s movements. Most of the biomechanical (including kinematic, kinetic and EMG) gait parameters improved over the starting condition, as well as clinical outcomes, although we also found some compensatory strategies.

### 3.1 Kinematic and kinetic results

**FIGURE 3:**
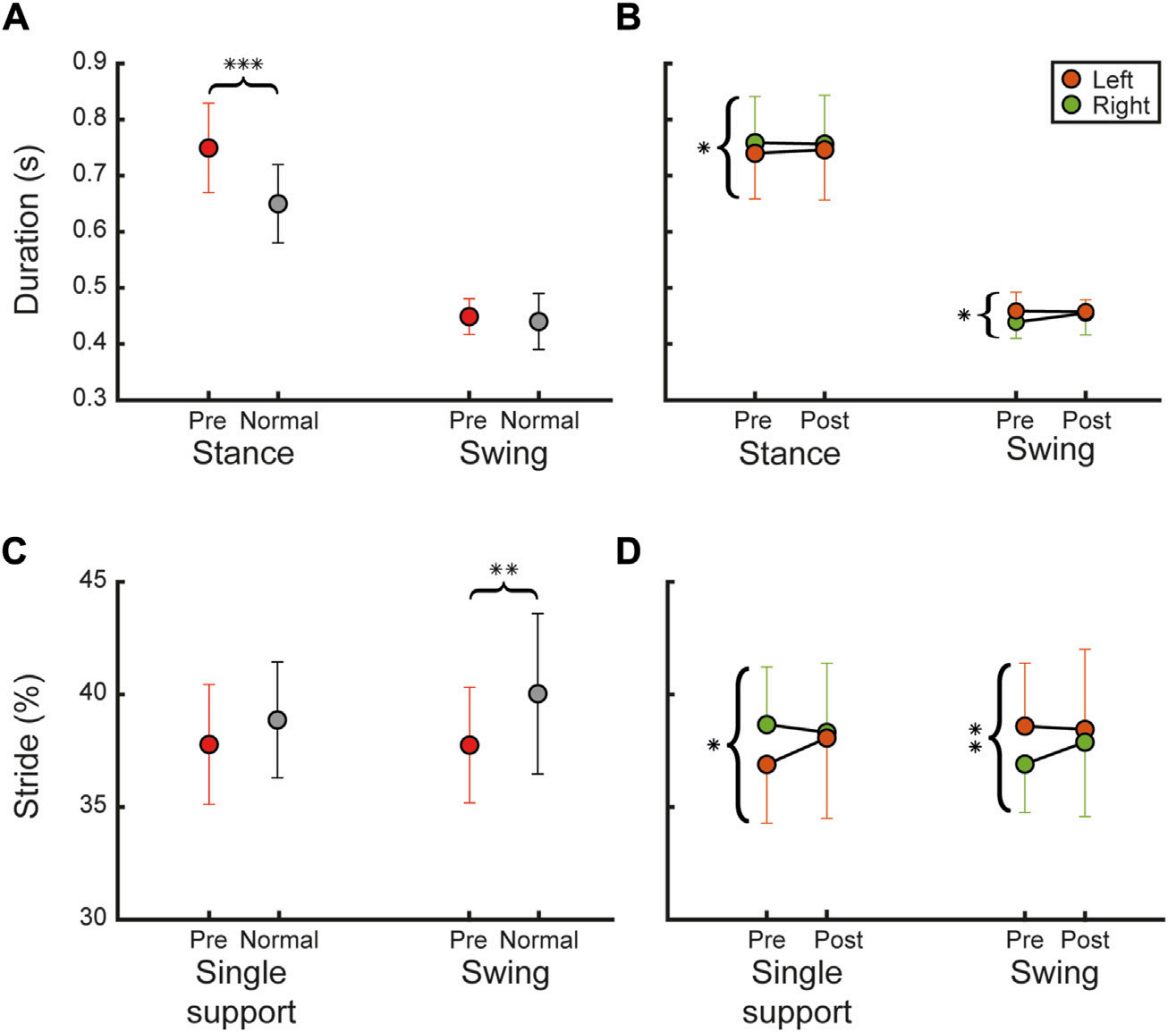
Temporal kinematic parameters. The figure reports temporal kinematic parameters as mean and SD overall participants. **(A)** Shows stance and swing duration between pre (red) and normal (grey) **(B)** shows comparison between pre- and post-intervention for left (orange) and right (green) lower limb **(C,D)** show the same comparisons as **(A,B)** respectively, for the temporal kinematic parameters normalized on the gait stride (gait stride single support and swing). Statistical difference significance is reported as *** = 0.001, ** = 0.01 and * = 0.05.

Regarding kinematic parameters, we analyzed spatial and temporal features of gait (see Table 3). We compared kinematic parameters of CA patients with normative data, at pre-intervention. In this regard, we found statistically significant difference in the duration of the stance phase between patients and normative values, evaluated at pre (*p* < 0.001) (see Figure 3A). In details, the symmetry between left and right at pre-invention was statically significant (*R* ^2^ = 0.97, *p* = 0.05) while no differences were observed at post intervention (*R* ^2^ = 0.96, *p* = 0.374) (see Figure 3B).

**TABLE 3:** Statistical comparisons of kinematic parameters. Coefficient of determination (*R* ^2^) and significance (*p*-value) of the LMM applied to the temporal and spatial gait parameters are reported in the table. Legend: RL (right limb), LL (left limb). Statistically significant *p*-values (*p* < 0.05) are shown in bold font.

		GAIT parameters	Pre VS normal	Pre gait symmetry (LL VS RL)	Post gait symmetry (LL VS RL)	Pre-post (LL)	Pre-post (RL)
Kinematic	Temporal	Stance duration (s)	***p* < 0.001**	*R* ^2^ = 0.97 ***p* = 0.050**	*R* ^2^ = 0.96 *p* = 0.374	*R* ^2^ = 0.46 *p* = 0.833	*R* ^2^ = 0.62 *p* = 0.924
		Swing duration (s)	*p* = 0.289	*R* ^2^ = 0.8 ***p* = 0.024**	*R* ^2^ = 0.43 *p* = 0.821	*R* ^2^ = 0.61 *p* = 0.886	*R* ^2^ = 0.41 *p* = 0.21
		Single support (%)	*p* = 0.122	*R* ^2^ = 0.69 ***p* = 0.038**	*R* ^2^ = 0.72 *p* = 0.789	*R* ^2^ = 0.29 *p* = 0.343	*R* ^2^ = 0.02 *p* = 0.787
		Swing (%)	***p* = 0.003**	*R* ^2^ = 0.86 ***p* = 0.010**	*R* ^2^ = 0.63 *p* = 0.592	*R* ^2^ = 0.01 *p* = 0.921	*R* ^2^ = 0.13 *p* = 0.416
		Gait velocity (m/s)	NA	NA	NA	*R* ^2^ = 0.71 *p* = 0.766	
	Spatial	Step width (m)	***p* < 0.001**	NA	NA	*R* ^2^ = 0.97 ***p* = 0.046**	

Regarding the swing duration, no statistical differences were observed between pre-intervention and normative values (see Figure 3A); however, the symmetry between left and right at pre-invention was statistically different (*R* ^2^ = 0.8, *p* = 0.024), contrary to post intervention (*R* ^2^ = 0.43, *p* = 0.821) (see Figure 3B).

In addition, we found that single support did not differ statistically from the normative values, at pre-intervention (see Figure 3C). However, the symmetry between left and right at pre-invention was statistically different (*R* ^2^ = 0.69, *p* = 0.038), contrary to post intervention (*R* ^2^ = 0.72, *p* = 0.789) (see Figure 3D).

The swing phase (%) evaluated at pre showed a statistically significant difference compared to normative values (*p* = 0.003) (see Figure 3C). In details, the symmetry between left and right at pre-invention was statically significant (*R* ^2^ = 0.86, *p* = 0.010), while no differences were observed at post intervention (*R* ^2^ = 0.63, *p* = 0.592) (see Figure 3D), suggesting an increased symmetry between two legs.

Furthermore, no differences were observed between gait velocity at pre (mean value ±SD = 0.688 ± 0.155 m/s) and post (mean value ±SD = 0.7 ± 0.141 m/s) between the two legs (*p* = 0.766). However, we found statistically significant differences between gait velocity of ataxic patients and normative values (mean value ±SD = 1.2 ± 0.2 m/s) at pre (*p* < 0.001), and at post (*p* < 0.001) for both lower limbs.

In addition, step width, which is the main compensatory mechanism in ataxic gait, differs statistically from the normative values at pre-intervention (*p* < 0.001). When comparing pre- (mean ±SD = 0.166 ± 0.052 m) to post-intervention (mean value ±SD = 0.155 ± 0.045 m) for both legs, a statistically significant difference emerged (*R* ^2^ = 0.97, *p* = 0.046).

Furthermore, we analysed other kinematic parameters of gait, which included pelvic obliquity, and trunk rotation angles. On the other hand, we analysed hip, knee and ankle moments and powers as kinetic values of gait (see Table 4 for more detail).

**TABLE 4:** Statistical comparisons of kinematic and kinetic parameters. Coefficient of determination (*R* ^2^) and significance (*p*-value) of the LMM applied to the spatial, joint power and ground reaction force gait parameters are reported in the table. The first column shows statistical comparison between R_CIRC_ of the cross-correlation and the relative delay with healthy adult normative values for left limb (LL) and right limb (RL) at pre and post respectively. Third and fourth columns show the comparison of the cross-correlation outcomes between each limb along evaluating sessions (pre VS post). Statistically significant *p*-values (*p* < 0.05) are shown in bold font.

		Gait parameters	pre gait symmetry (LL VS RL)	Post gait symmetry (LL VS RL)	Pre-post (LL)	Pre-post (RL)
Kinematic	Spatial	Trunk rotation (R_CIRC_)	*R* ^2^ = 0.42 ***p* = 0.004**	*R* ^2^ = 0.29 ***p* = 0.023**	*R* ^2^ = 0.92 ***p* = 0.025**	*R* ^2^ = 0.97 *p* = 0.949
		Pelvic obliquity (R_CIRC_)	*R* ^2^ = 0.23 *p* = 0.14	*R* ^2^ = 0.35 *p* = 0.28	*R* ^2^ = 0.5 ***p* = 0.014**	*R* ^2^ = 0.8 *p* = 0.42
Kinetic	Joint moments	Hip (R_CIRC_)	*R* ^2^ = 0.95 ***p* = 0.016**	*R* ^2^ = 0.59 *p* = 0.105	*R* ^2^ = 0.62 *p* = 0.732	*R* ^2^ = 0.85 *p* = 0.327
		Knee (R_CIRC_)	*R* ^2^ = 0.75 *p* = 0.763	*R* ^2^ = 0.88 ***p* = 0.036**	*R* ^2^ = 0.95 ***p* = 0.031**	*R* ^2^ = 0.13 *p* = 0.891
		Ankle (R_CIRC_)	*R* ^2^ = 0.01 *p* = 0.695	*R* ^2^ = 0.08 *p* = 0.554	*R* ^2^ = 0.04 *p* = 0.432	*R* ^2^ = 0.63 *p* = 0.638
	Joint powers	Hip (R_CIRC_)	*R* ^2^ = 0.77 *p* = 0.62	*R* ^2^ = 0.22 *p* = 0.435	*R* ^2^ = 0.49 *p* = 0.676	*R* ^2^ = 0.1 *p* = 0.512
		Knee (R_CIRC_)	*R* ^2^ = 0.85 *p* = 0.413	*R* ^2^ = 0.65 *p* = 0.956	*R* ^2^ = 0.21 *p* = 0.122	*R* ^2^ = 0.38 *p* = 0.265
		Ankle (R_CIRC_)	*R* ^2^ = 0.95 *p* = 0.163	*R* ^2^ = 0.87 ***p* = 0.008**	*R* ^2^ = 0.87 *p* = 0.919	*R* ^2^ = 0.67 *p* = 0.236
	Ground reaction forces	Ant-post force (R_CIRC_)	*R* ^2^ = 0.61 *p* = 0.058	*R* ^2^ = 0.19 *p* = 0.08	*R* ^2^ = 0.74 ***p* = 0.008**	*R* ^2^ = 0.53 *p* = 0.117
		Med-lat force (R_CIRC_)	*R* ^2^ = 0.75 ***p* = 0.033**	*R* ^2^ = 0.1 *p* = 0.639	*R* ^2^ = 0.26 *p* = 0.128	*R* ^2^ = 0.06 *p* = 0.665
		Vertical force (R_CIRC_)	*R* ^2^ = 0.25 *p* = 0.812	*R* ^2^ = 0.03 *p* = 0.475	*R* ^2^ = 0.24 *p* = 0.071	*R* ^2^ = 0.63 *p* = 0.204

**FIGURE 4:**
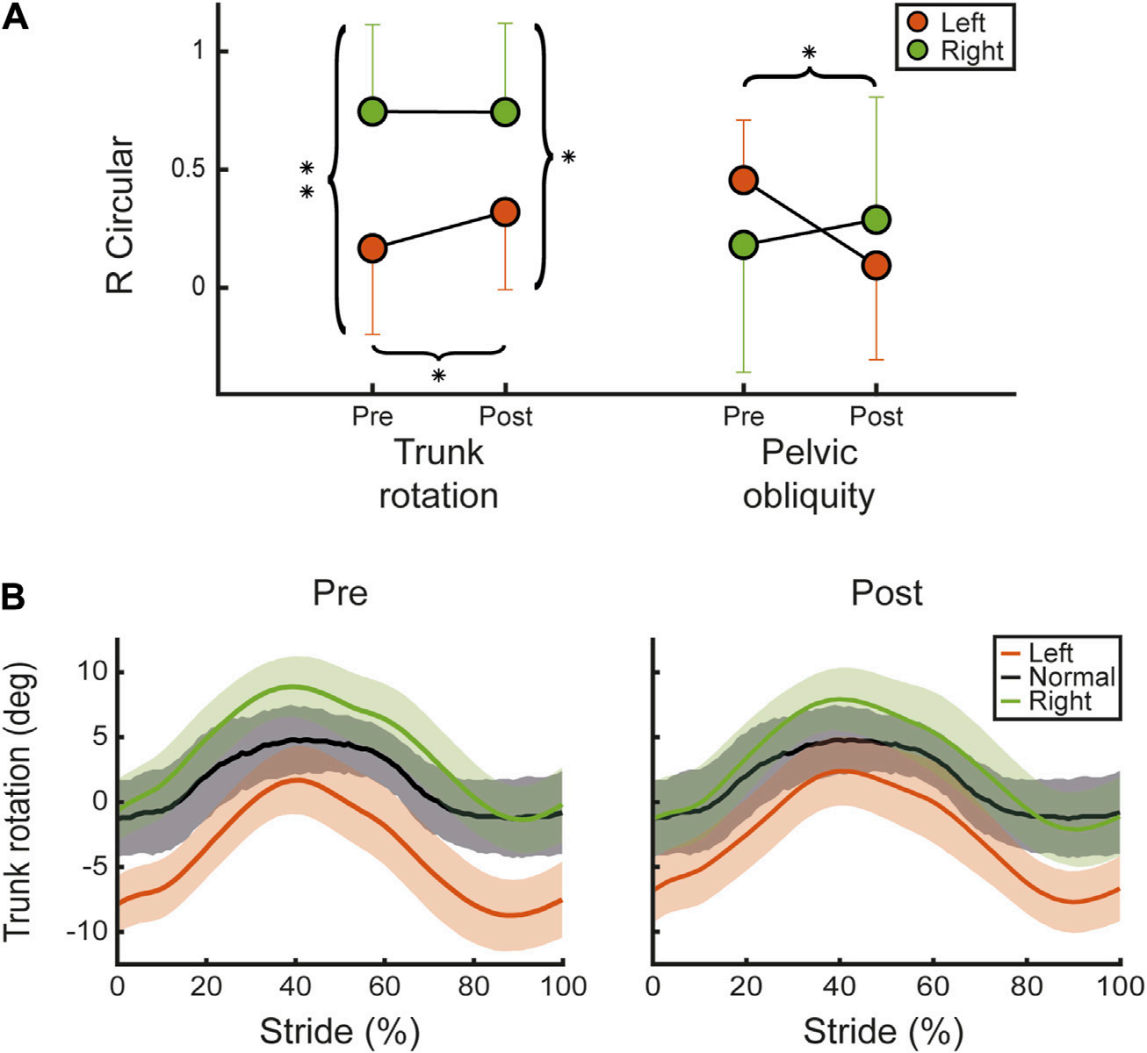
Kinematic angles. **(A)** Shows the mean and SD values overall participants for the shape symmetry index R_CIRC_ between the pelvic obliquity and trunk rotation angles recorded at pre and post for left (orange) and right (green) sides. **(B)** Shows the trunk rotation angle mean and standard deviation values overall participants, normalized on stride cycle, for pre (left panel) and post (right panel). Left (red) and right (green) are compared to normative values for healthy adult subjects (black). Statistical difference significance is reported as *** = 0.001, ** = 0.01 and * = 0.05.

We calculated the average symmetry index (R_CIRC_) across subjects for both trunk rotation and pelvic obliquity angles, assessed at pre- and post-intervention for each side (left in orange, right in green), as illustrated in Figure 4A.

After the CAREN treatment, there was a statistically significant improvement (*R* ^2^ = 0.92, *p* = 0.025), in R_CIRC_ index associated with trunk rotation compared to the baseline condition (pre-treatment) in the left limb, as shown in Figure 4A. In addition, we found a statistically significant difference between left and right at pre (*R* ^2^ = 0.42, *p* = 0.004) which was less significant at post (*R* ^2^ = 0.29, *p* = 0.023), suggesting that the symmetry increased after the VR treatment. However, we observed a statistically significant decrease in the R_CIRC_ of pelvic obliquity on the left side between pre- and post-treatment (*R* ^2^ = 0.5, *p* = 0.014). Trunk rotation angles are detailed and illustrated in Figure 4B, in which the averages across subjects’ angles for both legs (left in orange, right in green) are compared to the normative values (grey) for healthy adults at pre (left panel) and post (right panel).

**FIGURE 5:**
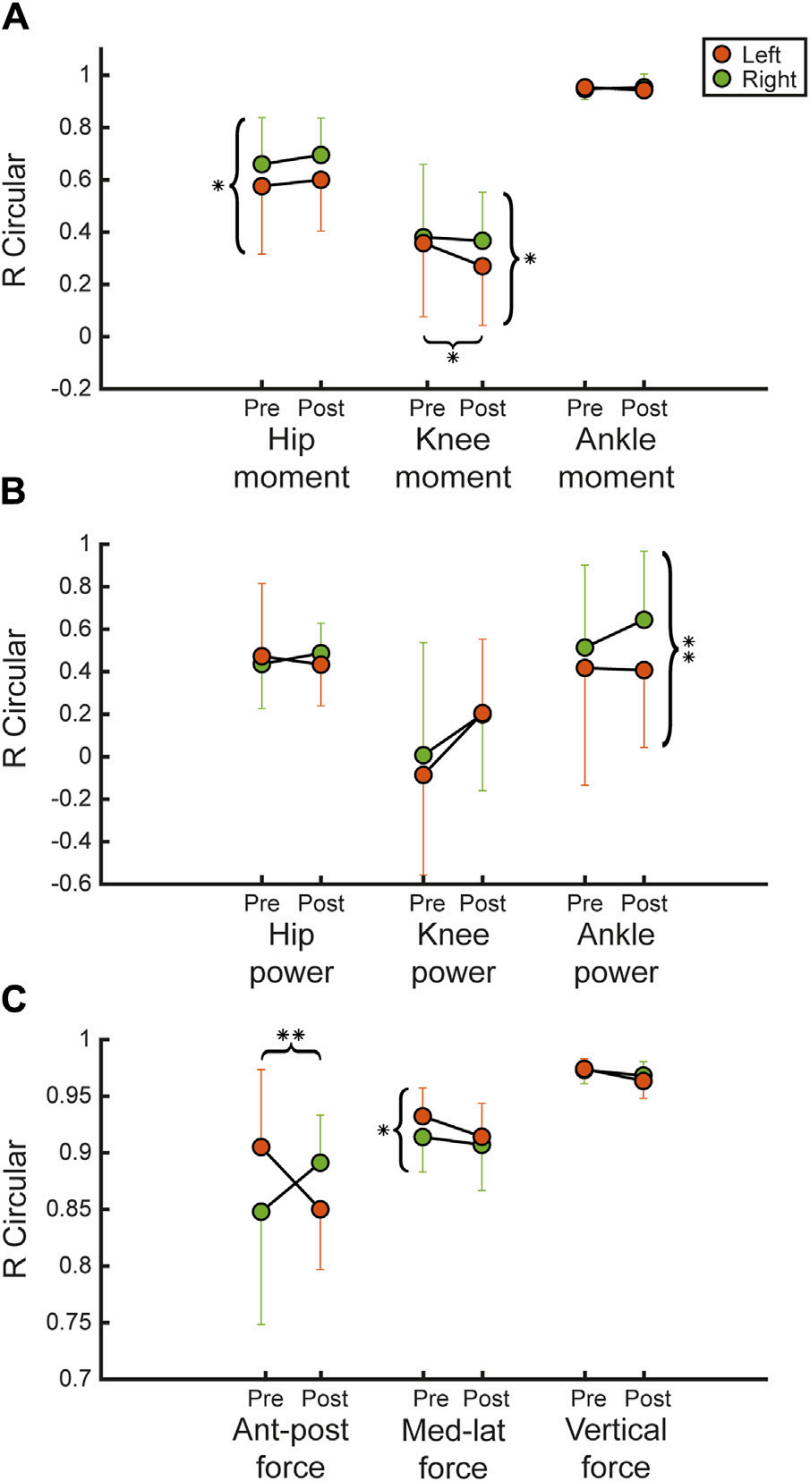
Kinetic parameters. Figure shows the mean and SD values overall participants of the R_CIRC_ index of the dynamic parameters. **(A,B)** show respectively hip, knee and ankle moments and powers, between pre and post for right and left lower limb. **(C)** shows R_CIRC_ index for the ground reaction forces (antero-posterior, medio-lateral, vertical strength) normalized on body weights. Statistical difference significance is reported as *** = 0.001, ** = 0.01 and * = 0.05.

Among the kinetic parameters of gait, we analyzed hip, knee and ankle moments between left and right limbs (see Figure 5A). We found that R_CIRC_ of hip moment showed a statistically significant difference between left and right at pre (*R* ^2^ = 0.95, *p* = 0.016) which is not present at post (*R* ^2^ = 0.59, *p* = 0.105). However, we found possible compensatory strategies detected after the CAREN intervention. In particular, the left limbs R_CIRC_ of the knee moment decreased statistically between pre and post (*R* ^2^ = 0.95, *p* = 0.031) and the symmetry between legs which was not statistically different at pre (*R* ^2^ = 0.75, *p* = 0.763), became different at post (*R* ^2^ = 0.88, *p* = 0.036). Regarding ankle power, we also noticed (Figure 5B) a significant difference at post (*R* ^2^ = 0.87, *p* = 0.008) between legs, which was not significant at pre (*R* ^2^ = 0.95, *p* = 0.163). In addition, we analysed ground reaction forces, including anterior-posterior, medio-lateral, and vertical forces. In detail, we found that there is a significant decrease in R_CIRC_ of the left limb in the antero-posterior plane at pre and at post (*R* ^2^ = 0.74, *p* = 0.008) (Figure 5C). Regarding medio-lateral forces, we also noticed a significant difference at pre (*R* ^2^ = 0.75, *p* = 0.033) between legs, which is not significant at post (*R* ^2^ = 0.1, *p* = 0.639), suggesting that the symmetry was increased respect at the beginning of the treatment.

### 3.2 EMG results

**FIGURE 6:**
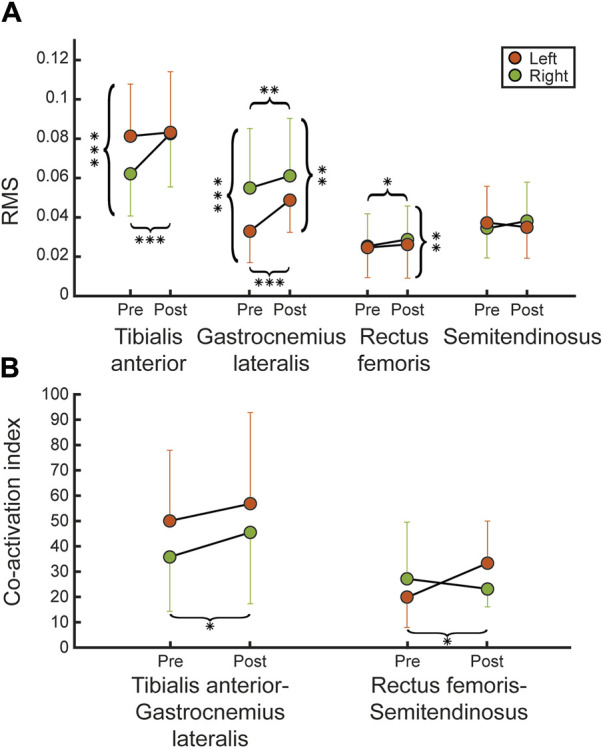
EMGs signal evaluations. **(A)** Shows the mean and SD values of the EMG signal evaluations (RMS) between pre and post for each recorded muscle (tibialis anterior, gastrocnemius lateralis, rectus femoris, semitendinosus) for left (orange) and right (green) legs, across all participants. **(B)** Shows the coactivation index reported for each agonist-antagonist muscle couple (tibialis anterior-gastrocnemius lateralis and rectus femoris-semitendinosus) for each leg. Statistical difference significance is reported as *** = 0.001, ** = 0.01 and * = 0.05.

To analyze muscle activity with EMG, we measured mean values and SD, and root mean square (RMS) overall subjects at pre and post, between left and right lower limbs, as shown in Figure 6A.

The RMS related to the tibialis anterior showed a statistically significant difference between left and right legs at pre (*R* ^2^ = 0.96, *p* < 0.001), which is not present at post (*R* ^2^ = 0.98, *p* = 0.782), suggesting an improvement in symmetry over the starting condition (pre-treatment). Regarding RMS related to rectus femoris, we found a significant difference, between two legs, at post (*R* ^2^ = 0.99, *p* = 0.008), which was not present at pre (*R* ^2^ = 0.98, *p* = 0.596), due to a significant increment between sessions for right limbs (*R* ^2^ = 0.98, *p* = 0.012). Regarding the co-activation index, we found a statistical significance of the right tibialis anterior-gastrocnemius lateralis (*R* ^2^ = 0.94, *p* = 0.024) and left rectus femoris-semitendinosus (*R* ^2^ = 0.39, *p* = 0.036) couples compared between pre and post (Figure 6B). Detailed data about RMS and coactivation index are reported in Table 5.

**TABLE 5:** Statistical comparisons of EMG analysis. Coefficient of determination (*R* ^2^) and significance (*p*-value) of the LMM applied to the RMS and the co-activation index of the EMG signals are reported in the table. The first and second columns show comparison between LL and RL respectively at pre and post. Third and fourth columns show the result of the comparison between each limb along evaluation sessions (pre VS post). Statistically significant *p*-values (*p* < 0.05) are shown in bold font.

		Muscle	pre (LL VS RL)	Post (LL VS RL)	Pre-post (LL)	Pre-post (RL)
EMG	RMS	Tibialis anterior	*R* ^2^ = 0.96 ***p* < 0.001**	*R* ^2^ = 0.98 *p* = 0.782	*R* ^2^ = 0.93 *p* = 0.643	*R* ^2^ = 0.98 ***p* < 0.001**
		Gastrocnemius lateralis	*R* ^2^ = 0.9 ***p* < 0.001**	*R* ^2^ = 0.89 ***p* = 0.01**	*R* ^2^ = 0.99 ***p* < 0.001**	*R* ^2^ = 0.99 ***p* = 0.002**
		Rectus femoris	*R* ^2^ = 0.98 *p* = 0.596	*R* ^2^ = 0.99 ***p* = 0.008**	*R* ^2^ = 0.99 *p* = 0.178	*R* ^2^ = 0.98 ***p* = 0.012**
		Semitendinosus	*R* ^2^ = 0.98 *p* = 0.094	*R* ^2^ = 0.97 *p* = 0.076	*R* ^2^ = 0.96 *p* = 0.254	*R* ^2^ = 0.97 *p* = 0.051
	Co-activation index	Tibialis anterior- Gastrocnemius lateralis	*R* ^2^ = 0.45 *p* = 0.132	*R* ^2^ = 0.16 *p* = 0.407	*R* ^2^ = 0.66 *p* = 0.479	*R* ^2^ = 0.94 ***p* = 0.024**
		Rectus femoris- Semitendinosus	*R* ^2^ = 0.56 *p* = 0.235	*R* ^2^ = 0.31 *p* = 0.07	*R* ^2^ = 0.39 ***p* = 0.036**	*R* ^2^ = 0.02 *p* = 0.612

Moreover, we have further analysed the comparison between affected vs. unaffected side for each gait parameter, including kinematic, kinetic and EMG signals (see Table 6).

**TABLE 6:** Statistical comparisons of instrumental gait parameters between affected vs. unaffected side**.** Coefficient of determination (*R* ^2^) and significance (*p*-value) of the LMM applied to the kinematic, kinetic, and EMG parameters are reported in the table. Legend: affected side (AS); unaffected side (US). Statistically significant *p*-values (*p* < 0.05) are shown in bold font.

		GAIT parameters	Pre VS normal	Pre gait symmetry (AS VS US)	Post gait symmetry (AS VS US)	Pre-post (AS)	Pre-post (US)
Kinematic	Temporal	Stance duration (s)	***p* < 0.001**	*R* ^2^ = 0.96 *p* = 0.42	*R* ^2^ = 0.96 *p* = 0.257	*R* ^2^ = 0.55 *p* = 1	*R* ^2^ = 0.53 *p* = 0.88
		Swing duration (s)	*p* = 0.289	*R* ^2^ = 0.56 *p* = 0.64	*R* ^2^ = 0.43 *p* = 0.821	*R* ^2^ = 0.67 *p* = 0.694	*R* ^2^ = 0.25 *p* = 0.381
		Single support (%)	*p* = 0.122	*R* ^2^ = 0.36 *p* = 0.935	*R* ^2^ = 0.73 *p* = 0.523	*R* ^2^ = 0.02 *p* = 0.565	*R* ^2^ = 0.32 *p* = 0.904
		Swing (%)	***p* = 0.003**	*R* ^2^ = 0.65 *p* = 0.386	*R* ^2^ = 0.66 *p* = 0.414	*R* ^2^ = 0.1 *p* = 0.758	*R* ^2^ = 0.01 *p* = 0.743
		Gait velocity (m/s)	NA	NA	NA	*R* ^2^ = 0.71 *p* = 0.766	
	Spatial	Step width (m)	***p* < 0.001**	NA	NA	*R* ^2^ = 0.97 ***p* = 0.046**	
		Trunk rotation (R_CIRC_)	NA	*R* ^2^ = 0.41 ***p* = 0.005**	*R* ^2^ = 0.34 ***p* = 0.013**	*R* ^2^ = 0.94 ***p* = 0.028**	*R* ^2^ = 0.94 *p* = 0.777
		Pelvic obliquity (R_CIRC_)	NA	*R* ^2^ = 0.01 *p* = 0.961	*R* ^2^ = 0.25 *p* = 0.587	*R* ^2^ = 0.25 *p* = 0.555	*R* ^2^ = 0.63 *p* = 0.316
Kinetic	Joint moments	Hip (R_CIRC_)	NA	*R* ^2^ = 0.93 *p* = 0.097	*R* ^2^ = 0.4 *p* = 0.536	*R* ^2^ = 0.91 *p* = 0.318	*R* ^2^ = 0.29 *p* = 0.794
		Knee (R_CIRC_)	NA	*R* ^2^ = 0.75 *p* = 0.996	*R* ^2^ = 0.77 *p* = 0.868	*R* ^2^ = 0.72 *p* = 0.541	*R* ^2^ = 0.52 *p* = 0.469
		Ankle (R_CIRC_)	NA	*R* ^2^ = 0.23 ***p* = 0.046**	*R* ^2^ = 0.31 *p* = 0.117	*R* ^2^ = 0.45 *p* = 0.957	*R* ^2^ = 0.01 *p* = 0.701
	Joint powers	Hip (R_CIRC_)	NA	*R* ^2^ = 0.78 *p* = 0.502	*R* ^2^ = 0.3 *p* = 0.253	*R* ^2^ = 0.72 *p* = 0.918	*R* ^2^ = 0 *p* = 0.823
		Knee (R_CIRC_)	NA	*R* ^2^ = 0.83 *p* = 0.775	*R* ^2^ = 0.65 *p* = 0.831	*R* ^2^ = 0.32 *p* = 0.157	*R* ^2^ = 0.24 *p* = 0.207
		Ankle (R_CIRC_)	NA	*R* ^2^ = 0.95 *p* = 0.233	*R* ^2^ = 0.64 *p* = 0.424	*R* ^2^ = 0.86 *p* = 0.746	*R* ^2^ = 0.79 *p* = 0.221
	Ground reaction forces	Ant-post force (R_CIRC_)	NA	*R* ^2^ = 0.34 *p* = 0.442	*R* ^2^ = 0.39 ***p* = 0.017**	*R* ^2^ = 0.31 *p* = 0.571	*R* ^2^ = 0.04 *p* = 0.775
		Med-lat force (R_CIRC_)	NA	*R* ^2^ = 0.51 *p* = 0.473	*R* ^2^ = 0.09 *p* = 0.694	*R* ^2^ = 0.6 *p* = 0.105	*R* ^2^ = 0.01 *p* = 0.692
		Vertical force (R_CIRC_)	NA	*R* ^2^ = 0.43 *p* = 0.204	*R* ^2^ = 0.18 *p* = 0.083	*R* ^2^ = 0.3 *p* = 0.06	*R* ^2^ = 0.33 *p* = 0.244
EMG	RMS	Tibialis anterior	NA	*R* ^2^ = 0.87 ***p* = 0.037**	*R* ^2^ = 0.91 ***p* = 0.002**	*R* ^2^ = 0.97 *p* = 0.645	*R* ^2^ = 0.95 ***p* < 0.001**
		Gastrocnemius lateralis	NA	*R* ^2^ = 0.99 ***p* < 0.001**	*R* ^2^ = 0.97 *p* = 0.173	*R* ^2^ = 0.98 ***p* < 0.001**	*R* ^2^ = 0.99 ***p* < 0.001**
		Rectus femoris	NA	*R* ^2^ = 0.97 ***p* = 0.006**	*R* ^2^ = 0.98 *p* = 0.144	*R* ^2^ = 0.98 ***p* = 0.004**	*R* ^2^ = 0.99 *p* = 0.328
		Semitendinosus	NA	*R* ^2^ = 0.99 *p* = 0.327	*R* ^2^ = 0.99 ***p* < 0.001**	*R* ^2^ = 0.98 **p = 0.044**	*R* ^2^ = 0.98 *p* = 0.102
	Co-activation index	Tibialis anterior-Gastrocnemius lateralis	NA	*R* ^2^ = 0.23 *p* = 0.603	*R* ^2^ = 0.12 *p* = 0.545	*R* ^2^ = 0.1 *p* = 0.36	*R* ^2^ = 0.9 *p* = 0.197
		Rectus femoris-Semitendinosus	NA	*R* ^2^ = 0.49 *p* = 0.469	*R* ^2^ = 0.01 *p* = 0.835	*R* ^2^ = 0.14 *p* = 0.208	*R* ^2^ = 0 *p* = 0.847

We observed that kinematic spatial-temporal parameters did not exhibit statistical differences, except for trunk rotation and step width, which displayed statistically significant differences similar to those observed in the left vs. right comparison. Similarly, kinetic parameters did not show statistical differences, except for ankle moment at pre-treatment (*R* ^2^ = 0.23, *p* = 0.046) and anterior-posterior force at post-treatment (*R* ^2^ = 0.39, *p* = 0.017), which were not evident in the left vs. right comparison. In addition, the EMG co-activation index did not show any statistically significant differences, although, some differences compared to the left vs. right analysis were observed in the RMS. Specifically, we observed a statistically significant difference between affected and unaffected sides in the rectus femoris, at pre-intervention (*R* ^2^ = 0.97, *p* = 0.006). Additionally, the tibialis anterior (*R* ^2^ = 0.91, *p* = 0.002) and semitendinosus (*R* ^2^ = 0.99, *p* < 0.001) displayed statistically significant differences at post-treatment, whereas no differences were observed for the gastrocnemius and rectus femoris muscles. Furthermore, we identified statistically significant differences in the rectus femoris (*R* ^2^ = 0.98, *p* = 0.004) and semitendinosus (*R* ^2^ = 0.98, *p* = 0.044) between pre- and post-rehabilitation treatment in the affected side (see Table 6).

### 3.3 Clinical results

**FIGURE 7:**
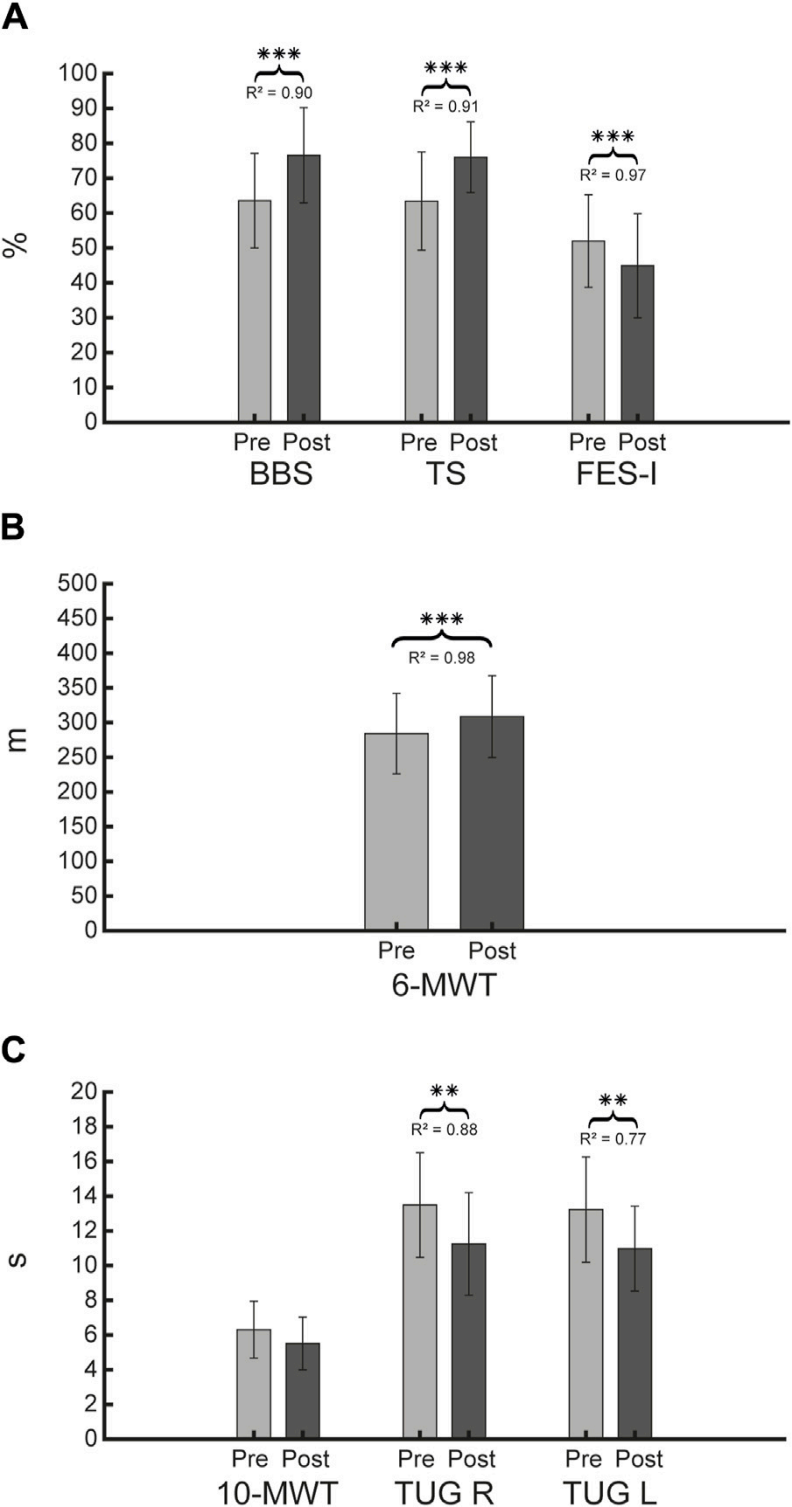
Clinical evaluation outcomes. Figure shows for each clinical evaluation scales the mean and SD, and *R* ^2^ for the LMM, respectively at pre and post for BBS, TS and FES-I **(A)**, 6-MWT **(B)**, 10-MWT, TUG R (right), and TUG L (left) **(C)**. Statistical difference significances are reported as *** = 0.001, ** = 0.01 and * = 0.05.

Moreover, we analysed clinical outcomes, regarding balance, gait functions, and fear of falls. In particular, we found statistically significances between pre and post for the following clinical outcomes BBS (*p* < 0.001), TS (*p* = 0.001), FES-I (*p* = 0.001) (see Figure 7A), 6-MWT (*p* = 0.001) (see Figure 7B), TUG L (*p* = 0.010), TUG R (*p* = 0.003) (see Figure 7C). In addition, we detected an improvement in 10-MWT, between pre- and post-intervention, without reaching statistical significance (*p* = 0.072) (see Figure 7C).

## 4 Discussion

To the best of our knowledge, this is the first study evaluating the effects and the feasibility of CAREN therapy in patients with CA. After 20 sessions of CAREN, kinematic, kinetic, and EMG parameters as well as clinical outcomes, such as balance (BBS, TS), and risk of falls (TUG, FES-I) were mostly improved, in CA patients. However, the most relevant result was found in trunk rotation during gait.

### 4.1 Kinematic parameters

We registered specific changes in the kinematic parameters after VR treatment, both in the spatial and temporal parameters. The results at post-treatment, regarding temporal parameters such as the duration of stance and swing, were more symmetrical when compared to pre-treatment. Additionally, we noticed that the duration of single support, in terms of stride cycle’s percentage, between the left and right limbs was more comparable between the two legs at post-treatment. These findings suggest an improvement in symmetry during gait. According to literature (Serrao and Conte, 2018; Bonanno et al., 2023; Cabaraux et al., 2023), patients with CA tend to show an asymmetric gait pattern, which is associated with imbalances. This is why it is important that CA patients can achieve a more symmetric gait. Our results can be explained by the fact that CAREN, with its 6-DOF platform, provides a series of triggers that improve lower limb loading, potentially leading to a more stable and safer gait (Lees et al., 2007). Despite these promising improvements, we did not observe an increase in gait speed. Since the patients were not given any information about the speed at which the exercises were performed, the increase in speed was not a useful indicator of gait recovery (Santucci et al., 2023). Our findings seem to suggest that our administered CAREN therapy is more effective in terms of gait stability, coordination and symmetry, rather than gait speed.

On the other hand, no improvements in the spatial parameters of the gait were observed in most cases, but a slight improvement in step width was registered. In healthy people, step width is about 8 cm (Skiadopoulos et al., 2020), whereas people affected by CA can have twice the stride width (Serrao and Conte, 2018). Our sample reduced their step width by one cm, suggesting that VR training with CAREN could also be useful for improving step width. As suggested by other authors, immersive VR environments, like CAREN, are more challenging for dynamic postural control, compared to real-world scenarios. In this sense, immersive VR can increase center of pressure displacement and motor responses (Renaux et al., 2022).

Generally, ataxic gait can show an amplified step width, recorded with gait analysis. In fact, step width is the main compensatory mechanism applied on the frontal plane, aiming to increase the safety distance between the center of mass and the edge of the stance surface (Serrao and Conte, 2018; Ilg et al., 2022; Cabaraux et al., 2023). As a result, patients with CA often show more changes in the temporal than in the spatial parameters of gait (Serrao and Conte, 2018), as also shown by our findings (Table 3). This suggests that irregularities in gait occur more frequently in the timing, coordination and duration of the different phases of gait than in the distance-related features of walking. This observation is consistent with the known role of the cerebellum in motor coordination and timing, as the cerebellum plays a crucial role in the fine-tuning of movements and ensures their smooth and coordinated execution (Ilg et al., 2007).

We also observed a substantial improvement in trunk rotation on the left side during gait, increasing the symmetry between the left and right sides. Regarding trunk rotation on the left side (R_CIRC_), we found a statistically significant difference between pre and post values, suggesting that trunk rotation at post-treatment was close to normative values. Trunk rotation around the vertical axis during gait is essential for maintaining balance, stability and efficiency of locomotion (Ceccato eta al., 2024). Besides this promising result, we also found a possible compensatory strategy, related to pelvic obliquity on the left side, which was closer to normal values, before the VR treatment. Generally, when trunk rotation improves during gait, it is synchronized with the movements of the lower limbs and pelvis (Ceccato et al., 2024). On the other hand, ataxic gait is characterised by incoordination between upper (trunk) and lower (legs) body parts, which results in increased upper body oscillations with a lack of local trunk stability (Castiglia et al., 2024). This aspect can cause an unstable, wide-base gait which is correlated with the progression of the disease, and it leads to impaired balance and risk of falling (Serrao and Conte, 2018). This is the reason why improving trunk rotation during gait in CA patients is fundamental (Freund and Stetts, 2010). It also has several benefits, such as reducing the energy cost of walking, increasing stride length and speed, and preventing injury or pain in the spine, hips and knees. According to Fleszar et al. (Fleszar et al., 2018), acoustic cues, which are also provided by VR rehabilitation training, could compensate for postural sway and imbalances. This aspect could suggest that VR treatment has led to a better balance during gait.

### 4.2 Kinetic parameters

The kinetic parameters included the ground reaction forces and the limb kinetic such as joint moments and powers.

We found that the hip moment R_CIRC_ of each lower limb was more symmetrical, after VR treatment. In particular, the hip moment during gait refers to the force developed by hip muscles and joint. Our results could suggest that the hip joint is moving in a more physiological manner when it is compared to normative values. This can have different effects depending on the phase of the gait stride, the type of gait and the individual characteristics of the person. For example, when walking, the hip joint generates force in two phases: at the end of the stance phase, when the hip extensors (such as the gluteus maximus) lean the body forwards, and at the beginning of the swing phase, when the hip flexors (such as the iliopsoas) lift the leg forwards (Sylvester et al., 2021). However, these findings at the hip joint could lead to a compensatory mechanism, due to pain/fatigue in the knee or ankle, since we noticed that knee moment on the left side was closer to the normative values, after the treatment. In addition, we found that ankle power values are less symmetric after treatment, although we noticed that the homogeneity of our sample increased, suggesting that some subjects achieved improvements in ankle power, without reaching statistical significance.

Furthermore, the mediolateral force was more symmetrical after the treatment, while the anterior-posterior and vertical forces were not different after treatment. This aspect means that the distribution of forces in side-to-side direction (mediolateral) is more evenly balanced between the left and the right foot during walking (Chockalingam et al., 2016). Otherwise, the distribution of forces in the forward-backward direction (anterior-posterior force) and the up-down direction (vertical force) are equally balanced. In our opinion, this result could be due to a variety of factors, such as better neuromuscular control, and/or compensatory mechanisms employed by ataxic patients to maintain balance and stability during gait (Chien et al., 2022). In the context of a VR rehabilitation program, ataxic patients may develop compensatory strategies to maintain balance and stability during gait (Chien et al., 2022). These strategies could result in a more symmetrical distribution of mediolateral (side-to-side) ground reaction forces.

### 4.3 EMG

Interestingly, we found an increase in muscle activations of the lower limbs, in the following muscles: rectus femoris, gastrocnemius lateralis and tibialis anterior bilaterally.

In particular, we found improvements after VR treatment in RMS for the following muscles: gastrocnemius lateralis and tibialis anterior, indicating an increase in the magnitude of muscle activation. Overall, the anterior tibialis plays a role in anticipatory postural adaptation. It favors knee flexion in the stance phase by causing a forward displacement of the tibia, while the gastrocnemius lateralis contributes to plantar flexion and knee flexion (Cardarelli et al., 2018; Farinelli et al., 2021). The increase in RMS in both muscles could indicate better control of the foot and more stability when walking. This aspect may have been promoted by VR training, which plays an important role in improving the knowledge of how to perform movements, leading to better overall neuromuscular control (Calabrò et al., 2017; Maggio et al., 2023).

Additionally, the improvement in lower limb muscle activation in CA patients is typically characterized as a positive change in patterns of muscle recruitment and coordination during movement (Mari et al., 2014). Improved muscle activation indicates that individuals with CA have achieved positive outcomes from the CAREN intervention. For example, improved muscle activation means better coordination of muscle contractions during the different phases of the gait cycle. The cerebellum plays a crucial role in coordinating movements, and VR interventions (Peri et al., 2019; Chien et al., 2022) such as CAREN may focus on improving this coordination to achieve smoother and more controlled lower limb movements. In addition, CA patients often develop compensatory strategies to maintain balance. Improved muscle activation indicates less reliance on compensatory movements.

Furthermore, we registered the co-activation index, a method to quantify the simultaneous activation of muscles during human movement (Souissi et al., 2017). This is the simultaneous activation of antagonist muscles that can stiffen joints and improve stability during limb movement, which is associated with high energy costs (Frey-Law and Avin, 2013). In the context of CA, studies have shown that patients exhibit increased co-activation of both ankle and knee muscles during the gait stride (Mari et al., 2014; Fiori et al., 2020). It is assumed that this increased coactivation or co-contraction is a compensatory strategy to reduce gait instability. The co-activation index in these patients is positively correlated with the severity of the disease and is more pronounced as the disease progresses, while it is negatively correlated with the risk of fall (Mari et al., 2014; Fiori et al., 2020). In our study, we observed increased co-activation between the gastrocnemius lateralis muscle (a calf muscle that supports plantar flexion and knee flexion) and the tibialis anterior muscle (a muscle that supports dorsiflexion and inversion of the foot), which often work in opposite directions to control ankle movement and stability. Our findings may suggest that this phenomenon may be a compensatory mechanism to improve stability during gait, as also evidenced by increased coactivation of rectus femoris and semitendinosus. In CA, gait is often unstable and uncoordinated due to damage to the cerebellum (Cabaraux et al., 2023). Increasing the co-activation of these muscles could therefore be an adaptive strategy to improve gait stability (Mari et al., 2014; Fiori et al., 2020; Li et al., 2021), as demonstrated also by instrumental kinematic parameters and clinical outcomes. However, it is noteworthy that improvements in muscle co-activation could lead to decreased joint mobility. This fact is in line with our findings, since we found changes in pelvic obliquity and knee moment on the left side, which were far from the normative values.

### 4.4 Clinical scales

Moreover, we found improvements in clinical outcomes such as balance (BBS, TS) and risk of falls (TUG, FES-I). In line with our findings, Peri et al. (Peri et al., 2019) showed similar results for balance outcome (BBS). The authors suggested that the use of VR environments is a realistic and highly motivating approach to treat CA patients, thus allowing long lasting training sessions. In particular, our results can be explained by the fact that VR training provides a multisensorial stimulation (e.g., through audio-video feedback), allowing an intensive, repetitive and task-oriented training, which is fundamental to boost neuroplastic processes (Calabrò et al., 2017; Maggio et al., 2023). In this way, CA patients receive augmented feedback to the central nervous system through the task performed in the virtual environment serving to develop the knowledge of the results of movements (knowledge of results) and the knowledge of the quality of movements (knowledge of performance), which are related to a training-specific motor learning and relearning (Calabrò et al., 2017; Maggio et al., 2023). Improvement in performance occurs when people can try out movements and are given feedback about the outcome of those movements so that they can then rectify or modify their performance to improve the outcome, as greatly provided by VR (Sharma et al., 2016). Unlike other VR devices, CAREN is an immersive virtual environment with a 6-DOF platform which provides a more realistic scenario (Lees et al., 2007). This aspect could have further contributed to improving coordination, and balance with a positive effect on fear of falling. Takimoto et al. (Takimoto et al., 2021) proposed that immersive VR training in CA patients can promote balance and functional recovery due to the provided sensory feedback directly in multiple forms, without any interference from the external environment. Therefore, this direct feedback fostered the feedforward learning for target movements better than conventional rehabilitation exercises. In addition, multi-sensory feedback and the repeated execution of motor tasks can improve the patient’s functional outcome, amplifying the processes of brain plasticity through motor learning, thus promoting cortical and subcortical modifications. It is noteworthy that immersive VR experiences, such as those provided by CAREN technology, have the potential to enhance motor recovery through top-down processes, rather than solely relying on bottom-up processes (i.e., sensory inputs transmitted to the brain). In this regard, the stimulation of mirror neurons, provided by visuomotor information in immersive VR, can facilitate the reorganization of damaged cortex, reduce cortical hyperexcitability, and promote neuroplastic processes such as dendritic spine formation and axonal sprouting. This enables the integration of perception, cognition, and action, as well as the retrieval of memorized motor plans. This process is attributed to reinforcement learning, fostering increased efficiency and improved performance (Maggio et al., 2023). Unlike any other type of immersive VR technology, the CAREN’s 6-DOF instrumented platform with the belt treadmill offers notable sensorimotor inputs to the brain, further enhancing the aforementioned stimuli. Hence, the benefit of employing VR, particularly with immersive technologies like CAREN, lies in generating a positive, enjoyable, and motivating learning environment for the patient, which demands individual control over multiple sensory-motor, cognitive, and social aspects (Calabrò et al., 2020).

Our study has some strengths, but also some limitations that need to be acknowledged. By or large, we observed a global improvement in gait functionality in patients affected by CA, suggesting that immersive VR environment could be a promising technology to treat this patient population. In addition, the use of instrumented gait analysis is another strength of our investigation, because it allowed the identification of those factors that should be considered during the treatment, including pelvic obliquity, knee moment and ankle power.

On the other hand, limitations are related to the generalizability of the results of the present work, which is currently limited by the small sample size and the heterogeneity of patients in terms of age (and then potential spontaneous and rehab-induced recovery). However, the pilot study involved a homogeneous group as per clinical syndrome; indeed, all patients have only CA and the associated mild hypoesthesia/hyposthenia in some of them did not affect rehabilitation and then our results.

Moreover, we further analysed the comparison between the affected vs. unaffected side for each gait parameter. Our findings indicate that the impaired side does not significantly influence the changes observed before and after the rehabilitation intervention, especially for temporal kinematic parameters. We attribute these results in part to the focus of our treatment, which was aimed at improving overall capabilities of gait and balance, not focusing only on the paretic side.

However, this pilot study needs to be confirmed by randomized controlled trials investigating these relevant changes and other important outcomes related to the efficacy of our innovative protocol. The absence of a control group prevents us from inferring any data on efficacy. Nonetheless, this is to be intended as a pilot study aimed at investigating the feasibility and potential effects of immersive VR in improving ataxia.

It is important to note that all patients were in the subacute/chronic phase of the disease (from 2 to 6 months after the event) and all of them were submitted to conventional physiotherapy alone before entering the study, and without important improvement on ataxic gait. Unfortunately, as they were referred to our laboratory from other centers, it was not possible to perform a clinical evaluation after the conventional physiotherapy alone, which would have better supported the potential role of VR in improving functional outcomes. Then, effectiveness of a combined VR training with CAREN plus physiotherapy with respect to traditional approaches must be deepened in the future by means of randomized controlled trials.

Nonetheless, in our previous cross-over work on PD patients (Calabrò et al., 2020), we have demonstrated that the functional outcomes were significantly higher after the CAREN than after conventional gait training. It is possible that the device may lead to similar results also in other neurological disorders like CA. Unfortunately, the cost of the device is extremely high, and this prevents its use in clinal practice, and it could be used to date only for research in specialized institutes. One may argue that the same results could be achieved by other less costly devices present on the market. However, in our opinion it is extremely unlikely that they can provide the same quality and amount of training and stimulation given the complexity and biomechanical features of the CAREN.

## 5 Conclusion

In this pilot study, we aimed to evaluate the feasibility of immersive VR training through CAREN in patients affected by CA. Our results suggested that CAREN may be useful to improve specific biomechanical parameters of gait, especially trunk rotation, in CA patients. It improved the quality of gait, making it stable, safe, and coordinated, thus reducing the risk of falls. However, more studies with larger samples are needed to extend our promising results to the global CA population.

## Data Availability

The raw data supporting the conclusion of this article will be made available by the authors, without undue reservation.
